# Prevalence of SPOP and IDH Gene Mutations in Prostate Cancer in a Jordanian Population

**DOI:** 10.1007/s10528-024-10974-4

**Published:** 2024-12-04

**Authors:** Mohammed S. Alorjani, Samir Al Bashir, Basmah Al-Zaareer, Sohaib Al-Khatib, Raed M. Al-Zoubi, Bahaa Al-Trad, Manal AbuAlarja, Ayman Alzu’bi, Mohammad Al-Hamad, Khalid Al-Batayneh, Mazhar S. Al-Zoubi

**Affiliations:** 1 Department of Pathology and Microbiology, Faculty of Medicine, Jordan University of Science and Technology, Irbid, Jordan; 2 Department of Biological Sciences, Faculty of Science, Yarmouk University, Irbid, 21163 Jordan; 3 Surgical Research Section, Department of Surgery, Hamad Medical Corporation, Doha, Qatar; 4 Department of Chemistry, Jordan University of Science and Technology, Irbid, 22110 Jordan; 5 Department of Biomedical Sciences, College of Health Sciences, Qatar University, 2713 Doha, Qatar; 6 Department of Basic Medical Sciences, Faculty of Medicine, Yarmouk University, Irbid, 21163 Jordan; 7 Department of Pathology, College of Medicine, Imam Abdulrahman Bin Faisal University (IAU), Dammam, Saudi Arabia

**Keywords:** SPOP, IDH1, Prostate cancer, Genetic mutation

## Abstract

Speckle-type POZ (SPOP) is described as an essential tumor suppressor factor in gastric cancer, colorectal cancer, and prostate cancer (PCa). *SPOP* gene mutations were reported in primary human PCa. Isocitrate dehydrogenase-1 (*IDH1*) oncogene mutations were detected in gliomas, acute myeloid leukemia, some benign and malignant cartilaginous tumors, and only 1% of PCa. This study aimed to investigate the prevalence of mutations of *SPOP* and *IDH1* genes in PCa in the Jordanian population. One hundred formalin-fixed paraffin-embedded tissue samples were collected from patients diagnosed with prostate adenocarcinoma. The obtained specimens were subjected to genomic DNA extraction, PCR amplification, and direct sequencing of exons 4, 5, 6, and 7 of *the SPOP* gene and exon 6 of the *IDH1* gene. *SPOP* gene mutations were found in 17% of PCa cases, while no mutation was detected in the screened exon 6 of the *IDH1* gene. Clinicopathological data demonstrated a strong correlation between prostate-specific antigen (PSA) levels and both Gleason score (GS) and the International Society of Urological Pathology (ISUP) grade group (GG). There was no significant correlation between PSA levels and age (*p* = 0.816) nor there were significant associations for *SPOP* mutational status with age (*p* = 0.659), PSA levels (*p* = 0.395), GS (*p* = 0.259), and ISUP GG (*p* = 0.424) in the tested population. The study found a strong correlation between PSA levels and both GS and ISUP GG. It also identified a high frequency (17%) of SPOP gene mutations in Jordanian Arab PCa patients, mainly in exon 7. No IDH1 mutations were detected in exon 6.

## Introduction

Prostate cancer (PCa) is the most frequently diagnosed non-cutaneous malignancy in men and the second leading cause of cancer-related death among men in the United States (Siegel et al. 2023). Globally, prostate cancer incidence is ranked the fourth type of cancer after breast, lung, and colorectal cancers and the second most frequent type of cancer in men (Sung, et al. 2020). Prostate cancer was found to be less common among men with a low human development index (HDI) (Cao et al. 2021). In Jordan, prostate cancer is the fourth most common cancer among males (7.6%), after colorectal cancer, lung cancer, and urinary bladder cancer, which account for (13.1%), (11.0%), and (9.2%), respectively. It is the third leading cause of cancer-related death in Jordanian males, as data on mortality due to cancer show that lung cancer is the most common accounting for 22.4% followed by small intestinal and colorectal cancer (11.4%) and prostate cancers (8.6%).

PCa is a silent, slow-growing disease that strikes older men. At the early stages, PCa is asymptomatic, but in rare cases, it can cause symptoms once it progresses to the metastatic stage (Sekhoacha et al. 2022; Barsouk et al. 2020). Prostate-specific antigen (PSA) is commonly used as a tumor marker for the screening of PCa. Depending on age, the overall health of the patient, stage, and grade of cancer, there are several treatment options (Al-Abdin and Al-Beeshi 2018). Many risk factors are linked to the development and progression of PCa, including age, race, family history, and dietary factors (Bhagirath, et al. 2019). Additionally, genetic predisposition has been proposed to be associated with the development of PCa. For instance, men who have a first-degree relative with PCa have double the risk of developing the tumor. Moreover, those with two first-degree relatives affected have a fivefold greater risk compared with men with no family history (Steinberg et al. 1990). Race is another important risk factor for the development of PCa. For instance, African American men have an age-adjusted incidence of about 200 per 100,000 men compared with 120 per 100,000 white men. Differences in death rates are even more dramatic, being around 44 per 100,000 for African Americans, versus 19 per 100,000 for whites. Asians, Pacific Islanders, Native Americans, and Hispanic men all have a lower incidence of prostate cancer than whites, but only Asians and Pacific Islanders have appreciably lower mortalities; 9 per 100,000 versus 19 per 100,000 for whites (Barry and Simmons 2017).

Many studies have been conducted to elucidate the molecular pathogenesis of PCa by investigating mutation in candidate genes. Speckle-type POZ (*SPOP*) gene encodes the SPOP protein which contains two main structural domains MATH and BTB (Hjorth-Jensen et al. 2018; Nagai et al. 1997). SPOP is involved in the regulation of multiple cellular processes, including androgen receptor-dependent signaling, cell cycle regulation, cell apoptosis, proliferation, and animal development (Hjorth-Jensen et al. 2018). SPOP has been described as a tumor suppressor factor in gastric, colorectal, and prostate cancers (Li et al. 2011; Geng et al. 2013; Kim et al. 2013). Another study has suggested a novel function for SPOP in the DNA damage response pathways (Zhang et al. 2014). Barbieri et al. reported *SPOP* gene somatic mutations in 6% to 15% of cancers of the prostate, stating that all those mutations are localized on the MATH domain (Barbieri et al. 2012). Another study also reported *SPOP* gene mutation in kidney cancer (Li et al. 2014).

Isocitrate dehydrogenase (IDH) is a dimer of two distinct small and large domains. It is a metabolic enzyme essential for the conversion of isocitrate to α-ketoglutarate (Yasutake et al. 2002; Prensner and Chinnaiyan 2011). The mutant form of IDH enzyme loses the ability to convert isocitrate to α-ketoglutarate. Mutations in the *IDH1* gene are common in gliomas, acute myeloid leukemia (AML), and a subset of benign and malignant cartilaginous tumors (Zhao et al. 2009; Amary et al. 2011). In PCa, however, they are found in only about 1% of cases and are associated with early age of onset and determine a subclass of the tumor (The Molecular Taxonomy of Primary Prostate Cancer 2015).

The current study aimed to investigate the prevalence of mutations of *SPOP* and *IDH1* genes in prostate cancer in the Jordanian Arab population and, eventually, to develop a molecular database for this cancer in Jordan.

## Materials and Methods

### Tumor Samples and Patients

This study was approved by the Institutional Review Board (IRB) at King Abdullah University Hospital (KAUH), Jordan University of Science and Technology, Irbid, Jordan. Formalin-fixed paraffin-embedded (FFPE) tissue samples from 100 prostate tumors were obtained from patients who underwent transurethral resection of the prostate (TURP) or radical prostatectomy (RP) with adenocarcinoma diagnosis from the archives of the Pathology Department at KAUH from 2008 to 2019.

### DNA Extraction

Genomic DNA was extracted from FFPE tissue samples using the ZYMO Research Quick-DNA FFPE Miniprep Kit (ZYMO Research, Irvine, CA, USA) by following a detailed protocol. Four to ten sections of FFPE tissue, each approximately 5–10 µm thick, were deparaffinized by immersing in 1 mL of xylene for three cycles, each lasting five minutes, to ensure thorough removal of paraffin. After each xylene treatment, samples were washed three times with absolute ethanol to remove residual xylene. The tissue was then digested by adding 20 µL of Proteinase K solution and 400 µL of Digestion Buffer to the sample, followed by overnight incubation with shaking at 55 °C to fully digest the tissue matrix. Following digestion, samples were incubated at 90 °C for 30 to 60 min to further facilitate tissue breakdown and DNA release. DNA was subsequently purified according to the steps outlined in the manufacturer's protocol, which included binding, washing, and eluting the DNA. All extracted DNA samples were initially stored at – 20 °C for short-term use. For long-term storage and preservation for future analyses, DNA samples were kept at – 80 °C.

### Polymerase Chain Reaction (PCR)

Specific primers were designed by primer3 software for amplification of the target sequences for targeting exons 4, 5, 6, and 7 of the *SPOP* gene according to the Ensemble genome browser (Table 1). The PCR reaction was performed in a total volume of 30µL, by using 5X-master-mix from (FIREPol, Tartu, Estonia), the PCR protocol involved an initial denaturation step at 95 °C for 5 min, followed by 40 cycles of denaturation at 95 °C for 30 s, annealing for 30 s as presented in (Table 1), and elongation at 72 °C for 45 s. A final elongation step at 72 °C for 10 min was completed to confirm the complete extension of the PCR products.

**Table 1:** Primers’ sequences used for amplification of *SPOP* gene

EXON #	Sequence		Tm ^O^C	Product size (base pair)
4	F	5ʹ-TTT TTC AGG TTG TCC ACT TCC-3 ʹ	60	276
	R	5ʹ -CCT GGC CTT CAT GGA AAT TA-3ʹ		
5	F	5ʹ-CCA GTT CTA TCA AAA TGG ATG C-3ʹ	56	298
	R	5ʹ-CGC AAA AAC CAG ATC AAA GC-3ʹ		
6	F	5ʹ-AGT TGT GGC TTT GAT CTG GT-3ʹ	57	230
	R	5ʹ-TCT GGG AAC TGC TAG TCT CA-3'		
7	F	5'-TCG TCT ATC TGC TGG CAA AAA-3ʹ	60	366
	R	5ʹ-GCT GCA GTT TGT TTT GTA GTT GAG-3ʹ		

### Agarose Gel Electrophoresis

The product of each PCR reaction was resolved on 2% agarose gel. A total of 10 μL of PCR product was loaded per well. Electrophoresis was performed at 100 V for one hour. DNA was visualized under a UV transilluminator. Fragment sizes of each PCR were determined by comparison with a 100-base pair (bp) DNA ladder (Intron, South Korea).

### Sequencing

PCR products were analyzed using Sanger sequencing by (Macrogen, South Korea) as an external service. The output DNA sequencing data were analyzed by the Snap gene and Mutation surveyor software.

### Statistical Analysis

The raw data were tabulated using Microsoft Excel (Microsoft Corporation). Statistical analyses were performed with GraphPad Prism 9 (GraphPad Software, Boston, USA). A *p* value threshold of 0.05 was considered to be statistically significant.

## Results

DNA was extracted from 100 FFPE prostate cancer tissue samples from 100 patients who underwent either TURP (*n* = 70) or RP (*n* = 30) and were diagnosed with adenocarcinoma at KAUH. The target exons were amplified and sequenced by the Sanger sequencing method. One patient was excluded from the study due to unsuccessful DNA sequencing. The clinicopathological parameters of the included 99 patients are summarized in Table 2.

**Table 2:** Clinicopathological parameters of the study population

Age (years)		
	70.73	39–90
Total PSA (ng/mL)	60.6	
Gleason score	Number or % of cases	
3 + 3	19	
3 + 4	27	
4 + 3	5	
4 + 4	8	
4 + 5	25	
5 + 4	6	
5 + 5	8	
No data	1	

*PSA* prostate-specific antigen

**Fig. 1:**
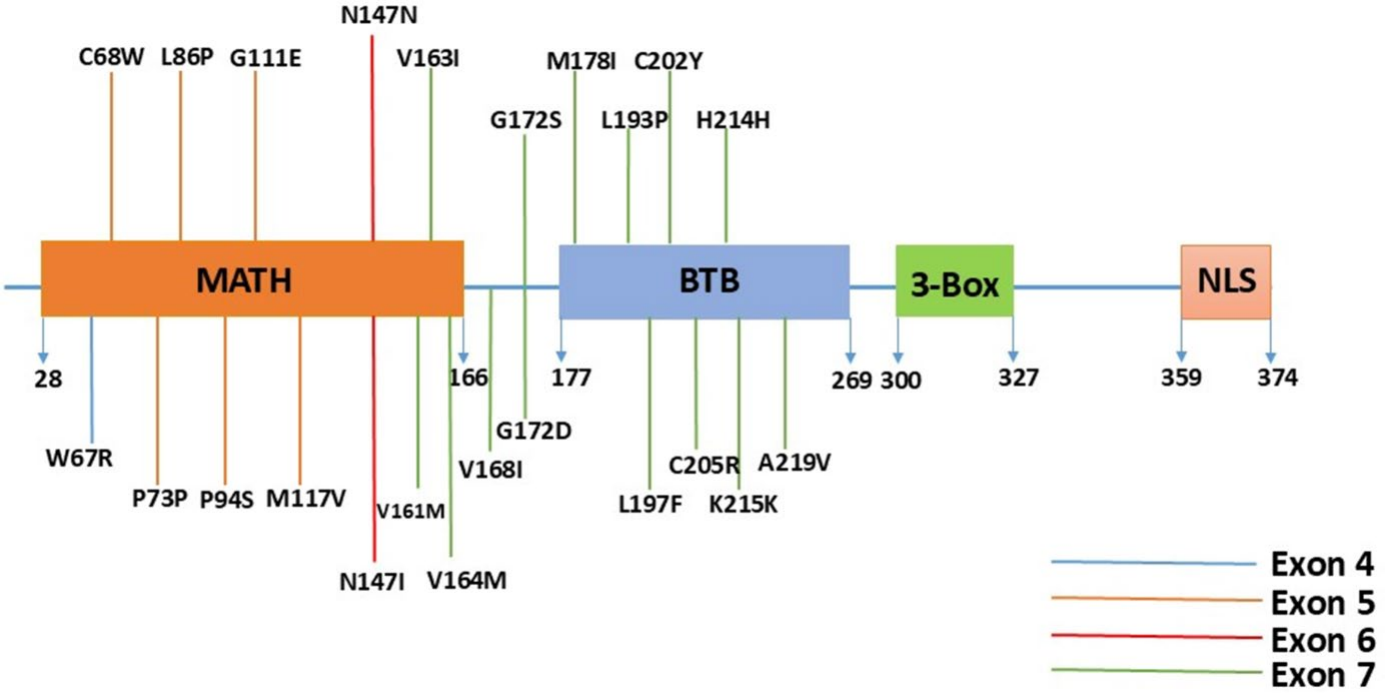
Illustration of the speckle-type POZ protein (SPOP) showing the different domains and the location of reported mutations in the PCa cases. (MATH: meprin and TRAF homology (MATH) domain) (BTB: a bric-a-brac, tramtrack and broad complex (BTB)/POZ domain) (NLS: C-terminal nuclear localization sequence). Genetic alterations in exons 4–7 are shown according to their location in the MATH and BTB domains

The current results showed the presence of a high prevalence of *SPOP* gene mutations, which are summarized in Table 3. The positions and distributions of the revealed *SPOP* gene mutations on MATH and BTB domains are clarified in Fig. 1. In particular, the results showed that two PCa patients aged 75 and 71 years old have a mutation at W67R in exon 4 of the *SPOP* gene. The total PSA levels for these two patients were 100 and 29.11 ng/mL, respectively, and the Gleason score (GS) gave a total score of 6.

**Table 3:** Mutations detected in the *SPOP* gene including age, PSA, and Gleason score

Sample#	Age (Years)	PSA (ng/ml)	Gleason score	SPOP Exon 4	SPOP Exon 5		SPOP Exon 6	SPOP Exon 7		
1	70	57.11	4 + 4		P94S	M117V				
2	73	10.28	3 + 3					G172S	C202Y	
3	49	77	5 + 4					V161M		
4	70	2.0	3 + 3					G172D		
5	81	536	4 + 5					A219V	H214H	
6	47	*	*		G111E					
7	75	100.8	*	W67R			N147N			
8	71	0.8	4 + 5					V163I		
9	70	2.15	4 + 4				N147I			
10	56	0.46	3 + 3					M178I		
11	56	1.13	3 + 3		C68W	P73P				
12	68	46.8	4 + 5					S197F		
13	77	49.8	4 + 5					L193P		
14	77	381.1	5 + 4					V164M	K215K	V168I
15	83	108	4 + 5					C205R		
16	79	0.34	3 + 3		L86P					
17	71	29.11	3 + 3	W67R						
Average	69	87.7		2	4	1	2	10	3	1

*PSA* prostate-specific antigen

On exon 5 of the *SPOP* gene, there were six different mutations distributed as five missense mutations C68W, L86P, P94S, G111E, M117V, and one silent mutation P73P. Interestingly, the C68W missense mutation and P73P silent mutation were found in the same patient who was 56 years old, with a PSA level of 1.13 ng/ml and a GS of 3 + 3 = 6. The patient with L86P mutation was 79 years old, with 0.13 ng/ml PSA level and a GS of 3 + 4 = 7. Another patient had a double mutation at P94S and M117V and was 70 years old with a PSA level of 57.11 ng/ml, and a GS of 4 + 4, giving a total score of 8 and International Society of Urological Pathology (ISUP) grade group (GG) 4. The patient with the G111E mutation was 47 years old with a high histologic grade.

Sequencing of exon 6 of the *SPOP* gene showed two mutations of the same codon, where a silent mutation N147N was found in a patient and a missense mutation N147I was detected in a 70-year-old patient with a GS of 4 + 4 = 8 and ISUP GG4.

Exon 7 showed the highest frequency of *SPOP* gene mutations in the study population. Fourteen different mutations, V161M, V163I, V164M, V168I, G172S, G172D, M178I, L193S, S197F, C202Y, C205R, H214H, K215K, and A219V, were detected in the tested PCa samples. The patient who had double mutations of G172S and C202Y was 73 years old with a 10.28 ng/mL PSA level and a GS of 3 + 3 = 6. The patient with V161M mutation was 49 years old, with 77 ng/mL PSA level and 5 + 4 = 9 GS and ISUP GG5. The patient with G172D mutation was 70 years old, with a GS of 3 + 3 = 6. Another patient who had a missense A219V mutation and a silent H214H mutation was 81 years old, with a PSA level of 536 ng/mL, 4 + 5 = 9 GS, and ISUP GG5. The patient with V163I mutation was 71 years old, with 4 + 5 = 9 GS and ISUP GG5. The patient with M178I mutation was 56 years old with a GS of 3 + 3 = 6. The patient with S197F was 68 years old, with 46.8 ng/mL PSA level, 4 + 5 = 9 GS, and ISUP GG5. The patient with L193P mutation was 77 years old, with 49.8 ng/mL PSA level, 5 + 4 = 9 GS, and ISUP GG5. The patient who had three mutations, V164M, V168I, and K215K silent mutation, was 77 years old, with PSA level of 381.1 ng/mL, GS of 5 + 4 = 9, and ISUP GG5. The patient with C205R mutation was 83 years old, with 108 ng/mL PSA level, 4 + 5 = 9 GS, and ISUP GG5. Finally, there was no mutation in the *IDH1* gene hotspot region in the Jordanian PCa cases.

**Fig. 2:**
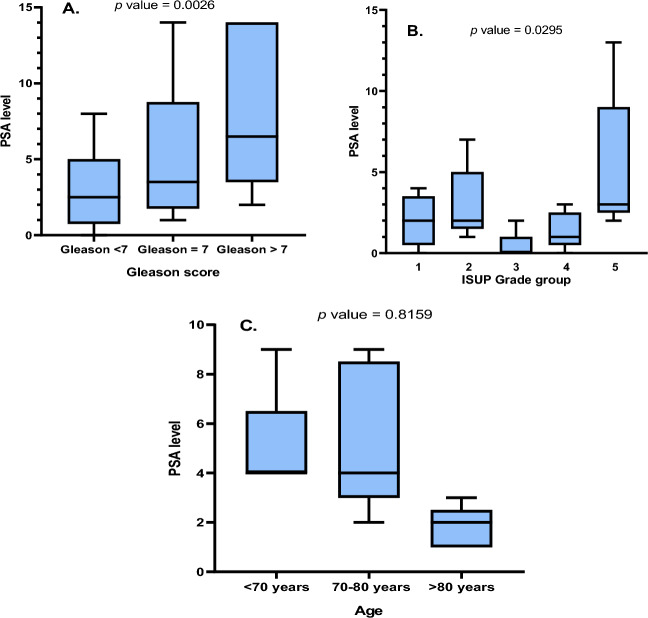
Correlation between PSA levels and Gleason score, ISUP grade group, and age. **c** Significant relationship between PSA levels and Gleason score. **b** Significant association between PSA levels and ISUP grade group. **c** No significant correlation between PSA levels and age. (PSA: Prostate-specific antigen) (ISUP: International Society of Urological Pathology)

The clinicopathological data demonstrated a strong and significant correlation between PSA levels and both GS and ISUP GG (*p* values: 0.0026 and 0.0295, respectively. Figure 2a and b). On the other hand, there was no significant correlation between PSA levels and age (*p *value: 0.8159. Figure 2c). However, age showed a tendency to lower PSA levels in individuals over 80 years old compared to the other age groups.

The clinicopathological characteristics, including age, PSA level, GS, and ISUP GG did not show significant associations with the mutational status in the tested population (*p* values > 0.05) as shown in Table 4.

**Table 4:** Clinicopathological characteristics for prostate cancer, comparing mutated and non-mutated cases

Variable	Range	Number		*p* value
		Mutated (*n* = 15)	Non mutated (*n* = 84)	
Age	Mean	Mean = 70.56	Mean = 71	0.6593
	< 70	5 (33.33%)	33 (39.75%)	
	70–80	8 (53.33%)	34 (40.96%)	
	> 80	2 (13.33%)	16 (19.27%)	
PSA level	Mean	Mean = 112.22	Mean = 47.3	0.3954
	< 4	2 (16.67%)	9 (20.83%)	
	4–10	2 (16.67%)	15 (27.08%)	
	10–20	0 (0%)	7 (14.58%)	
	20–100	5 (41.66%)	14 (27.08%)	
	≥ 100	3 (25%)	6 (10.41%)	
Gleason score	< 7	3 (21.42%)	12 (14.63%)	0.2592
	7	2 (14.28%)	30 (36.58%)	
	> 7	9 (64.28%)	40 (48.78%)	
ISUP grade group	1	3 (23.07%)	14 (17.28%)	0.4241
	2	1 (7.69%)	24 (29.63%)	
	3	0 (0.0%)	4 (4.94%)	
	4	2 (15.38%)	8 (9.87%)	
	5	7 (53.84%)	31 (38.27%)	

*PSA* prostate-specific antigen, *ISUP* International Society of Urological Pathology

## Discussion

The current study aimed to screen the genetic variations in *SPOP* and *IDH1* genes in association with the development of PCa in Jordan. Our results showed the presence of a high frequency of *SPOP* gene mutations in the PCa cases in the Jordanian population (17%). Twenty-three different mutations were found in the *SPOP* gene, which was distributed on MATH and BTB domains. On the other hand, the PCa cases did not show any mutation in the hotspot at exon 6 in the *IDH1* gene.

At the molecular level, the detected point mutations in the *SPOP* gene were found in vital domains that are responsible for different mechanisms. For instance, amino acids from 71–191 are required for nuclear localization and 186–217 are important for the homodimerization of SPOP. In our study, most of the detected mutations are located at these sites. In particular, seventeen patients showed the presence of twenty-three mutations on the screened exons. Ten PCa patients exhibited fourteen mutations in exon 7 representing 59% of the mutated patients. Moreover, four patients showed six mutations in exon 5 (23%). Two patients showed two mutations in exon 6 (12%); one of them showed a mutation in exon 4 too, and one patient showed only a mutation in exon 4 (6%).

The heterozygous mutation at M117V was detected in a sample that changes methionine to valine, which was previously described in cervical and endometrial cancer (Zehir et al. 2017; Gallo et al. 2017; Hoang et al. 2015; Jones et al. 2014). Another heterozygous mutation at G111E at exon 5 which changes glycine to tyrosine has been described by a previous study in prostate cancer (Buckles et al. 2014). The heterozygous mutation at V161M was detected and previously described in pancreatic cancer (Zehir et al. 2017). The homozygous mutation at S197F has been previously described in skin cancer (Krauthammer et al. 2015).

SPOP is composed of three basic domains: MATH; an N-terminal part containing residues 28–166, BTB; a C-terminal part containing residues 172–329 and a BACK domain, which assembles SPOP dimers into oligomers. The MATH domain plays a vital role in selectively recognizing, recruiting and mediating interaction with protein-ubiquitin ligase substrates, such as H2AFY and BMI1 (Zhuang et al. 2009). The BTB (POZ) domain mediates dimerization and interaction with CUL3 (Geersdaele et al. 2013). The BACK domain serves as a second place to mediate dimerization (Marzahn et al. 2016). Through these interactions, SPOP contributes to ubiquitination and protein degradation (Takahashi et al. 2002). SPOP regulates many proteins responsible for maintaining cell integrity (Zhang et al. 2015; Kwon et al. 2006), regulation of proteasome-mediated degradation of several oncoproteins and direct involvement in the DNA damage response, cellular signaling pathways, and cancer suppression (Wei et al. 2018). *SPOP* gene has been reported as a tumor suppressor in prostate cells by promoting the turnover of steroid receptor coactivator-3 (SRC3) protein through its ubiquitination, proteasomal degradation, and suppressing androgen receptor (AR) transcriptional activity (Geng et al. 2013).

Generally, certain genetic alterations commonly contribute to the downregulation of *SPOP* gene expression (Cheng et al. 2019). At the functional level, V163I and V164M mutations are located at the end of the MATH domain and almost in the junction between the MATH and BTB domains (Zhuang et al. 2009), while SCR-3 binding and androgen receptor activity can be affected by the mutation at G111E (Buckles et al. 2014). Any residue of amino acids located between 161 and 165 is expected to interfere with the dimer interface for the MATH domain (Yan et al. 2019). Collectively, the current results support the association of *SPOP* gene mutation with the development of PCa as shown in the previous findings in different studies in different populations (Blattner et al. 2014; Yoon et al. 2016; Hernandez-Llodra et al. 2019). Other studies have reported *SPOP* gene mutations in up to 15% of PCa (Barbieri et al. 2012).

On the other hand, the R132H hotspot mutation at exon 6 of the *IDH1* gene was not detected in PCa in the Jordanian population. Isocitrate dehydrogenase is present in different isoforms and has an essential role in cellular respiration in the tricarboxylic acid (TCA) cycle (Zhao et al. 2009; Fujii et al. 2016). IDH1 is a homo-dimeric cytoplasmic enzyme that reversibly converts isocitrate to α-ketoglutarate in the cytoplasm, presumably for the concomitant reduction of NADP+ to NADPH (Leonardi et al. 2012). The mutant enzyme loses the ability to convert isocitrate to α-ketoglutarate (Zhao et al. 2009). In addition, the enzyme gains a new function that leads to the accumulation of δ−2-hydroxyglutarate; the levels of which strongly correlate with tumorigenesis (Dang et al. 2016). Mutations in *the IDH1* gene are missense mutations occurring mostly at the residue R132 (Figueroa et al. 2010; Kang et al. 2009). Several cancer types are associated with mutations in *IDH1* gene, including glioma, paraganglioma, supratentorial primordial neuroectodermal tumor, acute myeloid leukemia, PCa, thyroid cancer, colon cancer, chondrosarcoma, cholangiocarcinoma, peripheral T-cell lymphoma, and melanoma (Zhao et al. 2009; Amary et al. 2011; The Molecular Taxonomy of Primary Prostate Cancer 2015; Abdel-Magid 2014). *IDH1* mutations occur in prostate cancer but at a very low frequency. About 0.3% of PCa have an *IDH1* R132H mutation and these are mostly heterogeneous (Hinsch et al. 2018). However, the current study did not recognize any mutation in exon 6 of *the IDH1* gene in the PCa cases in Jordan.

Furthermore, the current study explored the possible impact of the mutational status of the *SPOP* gene on the clinicopathological data of the cohort; however, there was no significant association between the occurrence of mutations in the *SPOP* gene and the PSA levels, GS or ISUP GG. Nevertheless, the PSA level was significantly associated with the GS and ISUP GG, which is inconsistent with a large-scale study in Stockholms (Palsdottir et al. 2019), while supporting previous findings of the association between PSA levels and Gleason score (Okolo et al. 2008). In some studies, PSA density has been suggested to understand the exact correlation between GS and PSA levels (Corcoran et al. 2012).

The main limitation of our study is that it is based on a relatively small sample size with some patient files having missing data (such as survival follow-up) needed for proper clinical correlation and survival analysis. However, this study, to the best of our knowledge, is the first to investigate the prevalence of mutations of the important genes, *SPOP* and *IDH1*, in PCa in Jordan. Moreover, it augments the efforts to develop a molecular profile database for this cancer in Jordan and the region. Furthermore, the aforementioned limitation can be overcome by engaging in collaborative research with other cancer centers in our country and the region.

## Conclusions

The current study confirmed the presence of a high frequency of *SPOP* gene mutations in PCa in the Jordanian Arab population; reported in four exons in 17% of the studied PCa patients, consistent with the findings of previous reports from other populations. Most of these mutations were found in exon 7 representing the hotspot exon in the PCa cases. On the other hand, none of the tested samples showed the presence of *IDH1* mutation in the screened exon 6 of the gene. Moreover, the results revealed a strong correlation between PSA levels and both GS and ISUP GG.

## Data Availability

No datasets were generated or analysed during the current study.
